# Free Cholesterol Induces Higher β-Sheet Content in Aβ Peptide Oligomers by Aromatic Interaction with Phe19

**DOI:** 10.1371/journal.pone.0046245

**Published:** 2012-09-25

**Authors:** Xiaolin Zhou, Jie Xu

**Affiliations:** 1 Department of Neurology, Shanghai First People’s Hospital, Shanghai Jiao-Tong University School of Medicine, Shanghai, China; 2 GI Division, Shanghai Jiao-Tong University School of Medicine Renji Hospital, Shanghai Institute of Digestive Disease, Shanghai, China; Cambridge Institute for Medical Research, United Kingdom

## Abstract

Accumulating experimental evidence support an enhancing effect of free cholesterol on amyloid-beta (Aβ) aggregation. To probe the mechanisms of cholesterol-mediated Aβ aggregation, we applied all-atom molecular dynamic simulations on Aβ42 peptides in presence of free cholesterol. Several control systems were also designed to examine the specificity of cholesterol-residue interactions, including mutation on aromatic residue, substitution of cholesterol with sphingomyelin (SM) and DPPC bilayer, and a mixing SM and cholesterol. Each system was performed 4 independent simulations, with a total time of 560 ns. It was found that cholesterol increased β-sheet formation by 4 folds, but the Phe19→Ser mutation on Aβ42 peptide totally eliminated cholesterol’s effect. A stable contact was recognized between the steroid group of cholesterol and the Benzyl group of Phe19. Interestingly, our simulation revealed a regular 1 ns time interval between the establishment of cholesterol-phenylalanine contact and consequent β-sheet formation, suggesting an important role of steroid-benzyl interaction in cholesterol-mediated aggregation. The presence of SM slightly increased β-sheet formation, but the mixture of cholesterol and SM had a strong induction effect. Also, the measurement of Phe19-lipid distance indicates that aromatic side chains of peptides prone to bind to cholesterol on the surface of the mixed micelle. In the DPPC system, polar chains were attracted to the surface of membrane, yielding moderate increase of β-sheet formation. These results shed light on the mechanism of cholesterol-mediated fibrillogenesis, and help to differentiate the effects of cholesterol and other lipids on β-sheet formation process.

## Introduction

Both epidemiological and experimental studies strongly implicate a role for cholesterol in the pathogenesis of Alzheimer’s disease (AD) [1]. Experiments proved that cerebral amyloid-beta (Aβ) generation is cholesterol dependent [2], and drastically lowered Aβ levels were achieved by treating guinea pigs with simvastatin, a clinically used cholesterol-lowering drug [3]. The exact mechanism about higher cholesterol level resulting in more amyloid aggregation is still unclear, but some hypotheses have been partially supported. Recent studies focused on the relationship between cholesterol level and amyloid precursor protein (APP) processing, trying to explain amyloidosis with elevated Aβ peptide generation [4]. Evidence suggests that amyloid precursor protein (APP) processing may preferentially occur in the cholesterol-rich regions of membranes known as lipid rafts [5], and a harsh reduction of membrane cholesterol may affect APP processing [6]. However, these experiments do not reveal a functional context explaining why the APP processing system responds in such a sensitive manner to cholesterol [7]. In addition, multiple experiments demonstrated that the alternation of cholesterol level doesn’t affect the activities of α, β [1], or γ secretase [8], which are critical in APP processing. Thus, more experiments are needed to prove the causal relationship between cholesterol level and APP processing activity.

Different from cholesterol in lipid rafts, increased free cholesterol in cytoplasm has been found to affect the aggregation of soluble Aβ peptides into tangle and fibrils. In vivo experiments showed that tangle-bearing neurons contain more free cholesterol than adjacent tangle-free neurons [9], and mass spectrometry analysis revealed significant increase of cholesterol concentration in senile plaques as compared to neuropil [10]. Moreover, increased cholesterol level in mouse brain induced intraneuronal accumulation of Aβ oligomers, which was associated with impaired memory [11]. In vitro studies have also demonstrated the potentiation effect of cholesterol on Aβ fibrillogenesis [12]. Using fluorescently-labelled lipids, Avdulov et al. showed that Aβ aggregates had a preferential binding for cholesterol rather than for phosphatidylcholine and fatty acids [13]. When Aβ42 peptide was incubated in the presence of aqueous suspensions of microcrystalline cholesterol and cholesteryl acetate, considerable potentiation of long smooth helical fibril formation occurred, compared to control samples containing the Aβ42 peptide alone [14]. Using transmitted electron microscopy, Harris further revealed that cholesterol micelles bound periodically to Aβ protofibrils and mature fibrils [15]. Interestingly, different derivatives of cholesterol have also been reported to promote protein aggregation. The ozonation product of cholesterol (ChSeco) was found to induce Aβ aggregation in a dose-dependent manner [16]. The inflammation-derived cholesterol 5,6-secosterol aldehydes induced the misfolding of wild-type p53 into an amyloidogenic form that binds thioflavin T [17]. In summary, the above-mentioned studies suggest the steroid structure of cholesterol may play an important role in the formation of protein aggregates.

Due to limitations of current experimental techniques, a detailed knowledge of cholesterol-mediated fibrillogenesis at the atomic level is missing. Recent molecular dynamics studies shed light on assembly dynamics of shorter amyloid peptides [18], [19], [20], [21], [22] and conformational transition of full Aβ peptides [23], [24]. Interestingly, both experiments [25] and MD simulations [26] proved that the mutation Phe19→Ser significantly affect the folding and assembly of Aβ peptides. It was thus proposed that interactions between aromatic side chains might not only make a strong contribution to the thermodynamic stability of the amyloid structures but also provide order and directionality in the self-assembly [18].

The important role of aromatic residues in amyloid formation may give a clue on the mechanism of cholesterol-Aβ peptide interaction. We hypothesed that cholesterol’s steroid group bind to aromatic side chains of Aβ peptides and help to overcome the energy and entropy barriers to form β-sheet structure. In order to validate this hypothesis, we applied molecular dynamics simulations on Aβ peptide in presence of free cholesterol. Several control systems were also designed to probe the features of cholesterol-peptide interaction, including Phe19→Ser mutation, substitution of cholesterol with sphingomyelin (SM), DPPC bilayer, and a mixture of SM and cholesterol. The conformational transitions of Aβ peptides in each system were analyzed to evaluate the effect of different mutations and lipids.

**Table 1:** System composition except SPC water.

System names	Peptides	Cholesterol	Sphingomyelin	DPPC
Simple system	Aβ42 × 4			
Free Cholesterol	Aβ42 × 4	12		
Cholesterol & Mutant	Phe19→Ser × 4	12		
Context Mutation	Phe19→Ser × 4	12		
Sphingomyelin	Aβ42 × 4		12	
Cholesterol & Sphingomyelin	Aβ42 × 4	12	12	
Membrane	Aβ42 × 4			128

## Materials and Methods

### Structural Templates

Initial coordinates of wild-type Aβ42 peptide was taken from an aqueous solution structure determined by NMR [24], (PDB file: 1Z0Q). We simulated the peptide in water for 100 ns to get a random coil conformation, which was then used to construct wild-type Aβ42 peptide systems. To prove the specificity of steroid-benzyl interaction, we made Phe19→Ser mutation on the wild-type Aβ42 peptide, and the resultant structure was minimized before MD simulation with free cholesterol.

The structure of cholesterol was a part of a cryptogein-cholesterol complex, which was determind by x-ray diffraction in 1.45 A resolution [27], (PDB file: 1LRI).The sphingomyelin structure was provided by the Klotho Biochemical Compounds Declarative Database [28]. We used a fully hydrated, equilibrated membrane phospholipid bilayer containing 128 DPPC molecules from Tieleman [29]. The simple point charge (SPC) water model was used to solvate all the systems.

### Simulation Systems

Seven different systems were constructed to investigate the features of interactions between free cholesterol and Aβ42 peptides. The compositions of all systems have been shown in **Table 1**.

All systems were solvated using SPC water, and other components have been described as following: Simple system consists 4 Aβ42 peptides; Free cholesterol system includes 4 Aβ42 peptides and 12 separated cholesterol molecules; Cholesterol and mutant system contains four Aβ42 mutants (Phe19→Ser) and 12 separated cholesterol molecules; The context mutation system was based on the 20-ns simulation result of free cholesterol system. After cholesterol bind with Phe19 residue and a local β-sheet was formed, we mutated Phe19 to Ser, and continued to simulate for another 20 ns; Sphingomyelin system consists 4 Aβ42 peptides and 12 separated sphingomyelin molecules; Cholesterol and sphingomyelin system is a mixture of 12 cholesterol and 12 sphingomyelin molecules plus 4 Aβ42 peptides; Membrane system has 4 Aβ42 peptides and a lipid bilayer consisting 128 dipalmitoyl phosphatidylcholine (DPPC) molecules.

**Figure 1:**
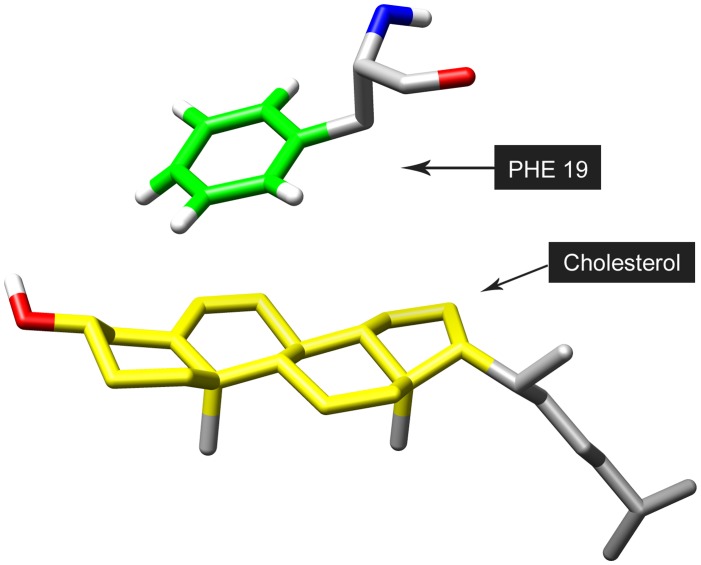
Definition of distance between Phe19 and cholesterol molecule. The contact is defined as the minimum distance between the benzyl group (in green) of Phe19 and steroid group (in yellow) of cholesterol.

### Simulation Protocol

MD simulations were performed using the GROMACS 3.3.1 package [30] with a modified version of GROMACS force field. Lipid force field parameters were used as described by Berger et al. [31] and Marrink et al. [32]. The topology parameters of cholesterol, sphingomyelin, and DPPC molecules for Groamcs force field were generated by the Dundee PRODRG2 Server [33]. Simulations were carried out in the NPT ensemble, with periodic boundary conditions. The initial velocities were taken randomly from a Maxwellian distribution at 300 K. The temperature was held constant by Berendsen coupling [34]. A constant pressure of 1 bar was applied with a coupling constant of 1.0 ps [35].The Van der Waals cutoff was set to 0.8 nm, and Long-range electrostatic interactions were calculated using the particle mesh Ewald summation methods [36] with a cut off of 1.4 nm. The pair lists were updated every 10 steps. The LINCS algorithm [37] was used to constrain bond lengths.

During energy minimization the steepest descents algorithm was used and the minima was reached in 500 steps. MD was performed with a time step of 2 fs and the coordinates were saved every 500 steps. Each system was simulated for 4 circles with from different starting coordinates. We set the simulation time for each circle to 20 ns, and used the resultant trajectories for further analysis. All the simulations were run on a HP XC Cluster Platform composed of 256 CPUs.

Secondary structure content was calculated using DSSP [38]. The extent of contact between cholesterol and residue Phe19, as shown in **Figure 1**, was modeled with the minimum distance between the Benzyl group of Phenylalanine and the steroid group of cholesterol molecule. Molecular graphics images were prepared using VMD [39].

## Results

### Cholesterol Increases β-sheet Formation

**Figure 2:**
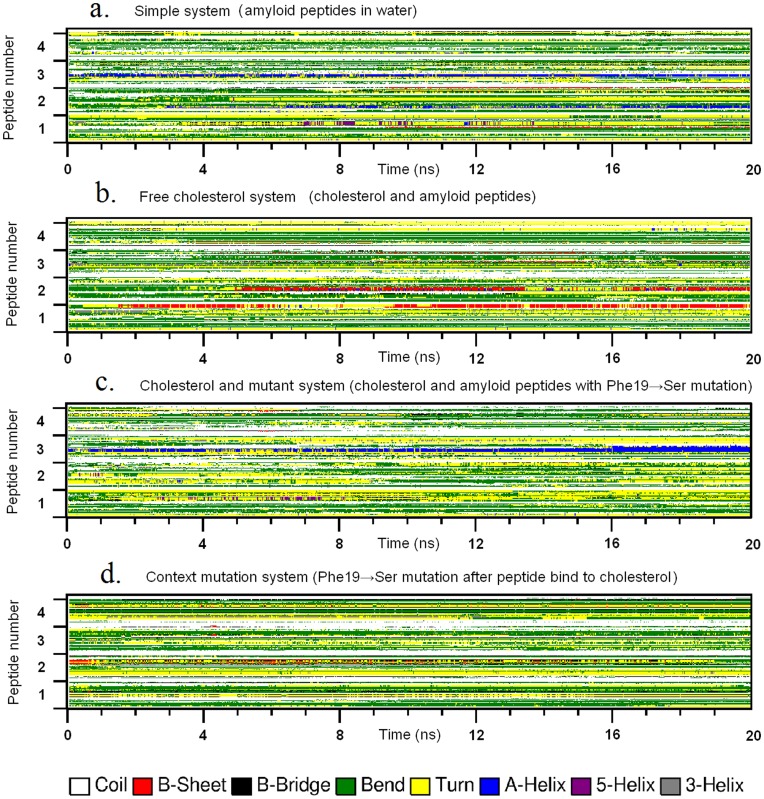
The conformational transition of peptides in simple system (a), free cholesterol system (b), cholesterol & mutant system (c) and context mutation system (d). The plots show the secondary structures of four peptides (axis y) in each system as a function of time (axis x). Secondary structures are drawn in different colors, with β-sheet structure in red.

In all simulation systems, the four Aβ peptides associated with each after a short period of about 2 ns. In the simple system (four Aβ peptides in water), the secondary structures were mainly random coil, bend, turn, and helix (as shown in **Figure 2a**). But after a time period of about 9 ns, a very short β-sheet structure began to form in the C-terminal. Once formed, the β-sheet was quite stable and existed to the end of the simulation. Comparing all the simulation systems, the frequencies of β-sheet formation of Aβ peptides were quite different. We summarized the frequency of β-sheet formation in all systems and got a relative ratio of 1.0 : 4.9 : 0.1 : 0.8 : 1.7 : 5.5 : 2.3 (simple system: free cholesterol: mutation: context mutation: sphingomyelin: cholesterol and sphingomyelin: DPPC bilayer system).

We analyzed the secondary structures of Aβ peptides in free cholesterol system (four Aβ peptides with 12 cholesterol molecules), and found that cholesterol significantly increased the β-sheet formation in the C-terminal fragments of peptides. As shown in **Figure 2b**, the β-sheet structures formed earlier, existed longer, and involved more residues and peptides. The first and second β-sheet structures appeared respectively after 1.5 and 5 ns, and the third one, which was very short, appeared at 4 ns. Notably, all these β-sheet structures formed earlier than those in the simple system. In addition, the frequency of β-sheet formation was 4-fold higher than that in simple system. These all strongly suggest cholesterol’s enhancement on β-sheet formation of Aβ peptide.

### Mutation on Aromatic side Chain Eliminates Cholesterol’s Effect

In order to validate the hypothesis that cholesterol enhances β-sheet by interacting with aromatic side chain, we made a Phe19→Ser mutation on the Aβ42 peptide. This configuration changed the aromatic group of Phenylalanine in position 19 into a polar side chain of Serine. We designed two kinds of starting conformations: one used well-separated molecules (namely mutant system); and the context mutant system was based on the simulated conformation of free cholesterol system. After the 20-ns simulation of cholesterol system, cholesterol formed stable contact with Phe19, and a stable β-sheet was also established within the Aβ peptide. Then we made Phe19→Ser mutation on this structure. The mutant system was minimized for 500 steps, followed by MD simulation of 20 ns.

As shown in **Figure 2c**, the mutation of Phe19 dramatically decreased β-sheet structure formation. Instead, more helix structures were formed during the later period of simulation. Although free cholesterol still existed in this system, its enhancement effect on β-sheet structure was totally eliminated. For the context mutant system (**Figure 2d**), the β-sheet structures formed in free cholesterol system gradually disappeared in the end of the simulation.

### Beta Sheet Formation after Steroid-benzyl Contact

**Figure 3:**
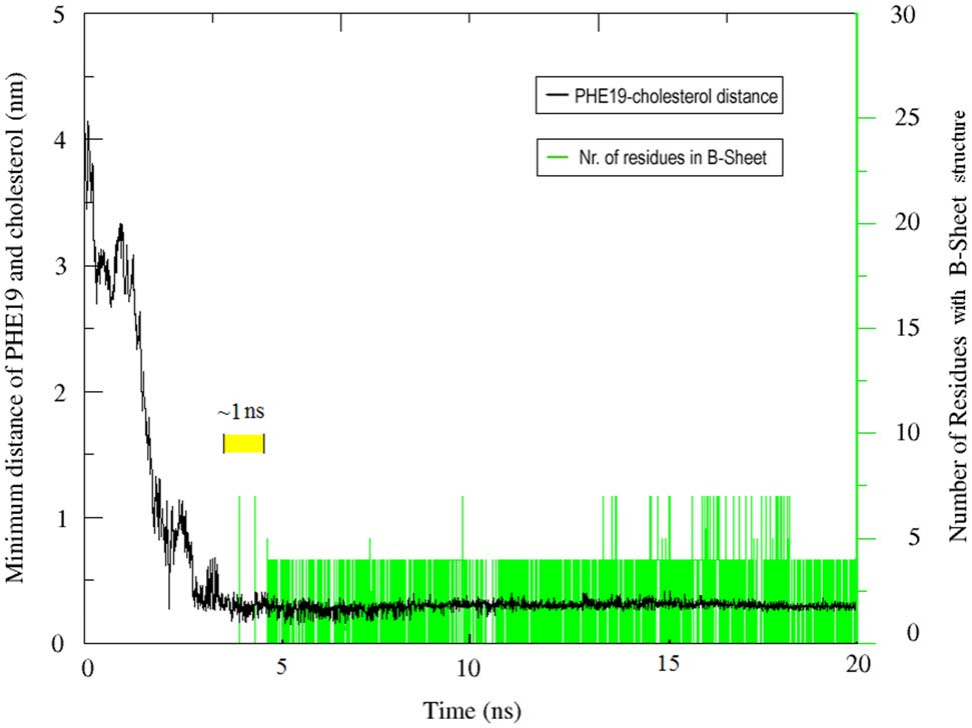
Time correlation of two events. The plot and axis in black show the distance of Phe19 and cholesterol as a function of time, while the plot and axis in green indicate the number of residues with β-sheet structure at the corresponding time points. Note the time interval of 1 ns (in yellow) between the contact of cholesterol-Phe19 and the formation of β-sheet structure.

To further investigate the mechanism of cholesterol-mediated β-sheet formation, we analyzed the relationship between cholesterol- Phenylalanine interaction and β-sheet formation. As described in the Methods section, we defined the extent of cholesterol-Phe19 contact with the minimum distance between the steroid group of cholesterol and the Benzyl group of Phe19. Our data suggest the contact between cholesterol and phenylalanine was rather stable, with the distance between benzyl group and steroid group of about 0.3 nm (**Figure 3**). More importantly, we found that β-sheet structures formed shortly after the establishment of cholesterol-Phe19 contact. After analyzing several trajectories, we found a regular time interval of about 1 ns between cholesterol-Phe19 binding and β-sheet formation. These sequential events strongly suggest a role of steroid-benzyl interaction in cholesterol-mediated β-sheet formation.

### Molecular Details of Cholesterol’s Induction Effect

**Figure 4:**
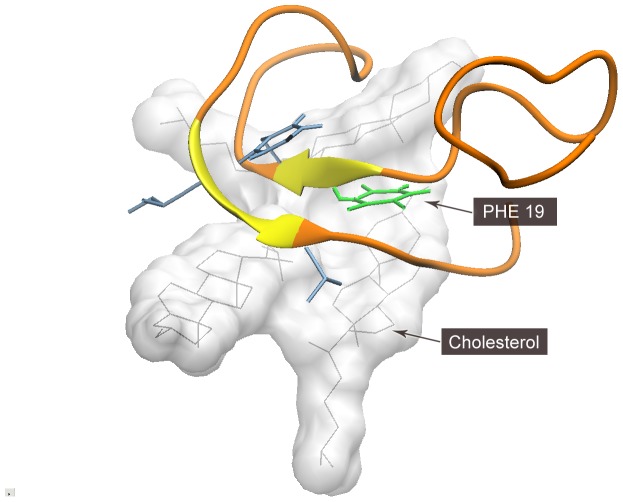
Interaction of cholesterol with Aβ peptide. It can be found that cholesterol (in white) forms a flat surface, attracting Phe19 (in green) to its surface via steroid-benzyl affinity. The adjacent hydrophobic residues (in blue) also contributed to the formation of β-sheet structure on the surface of free cholesterol.

To understand the detailed mechanism of cholesterol-Aβ peptide interaction, we observed the trajectories of Phe19 and its adjacent cholesterol molecules (**Figure 4**). An interesting finding is that cholesterol molecules assembled into a sheet-like template, and attracted Aβ peptide to its surface via benzyl group-steroid group affinity.

**Figure 5:**
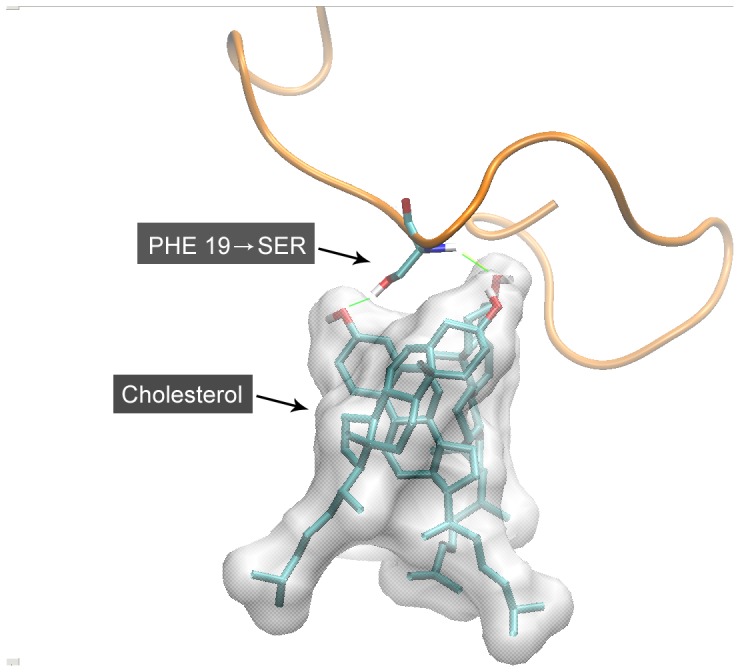
Interaction between cholesterol and mutant Ser19. The hydrogen atoms in amine group and alcohol group of serine formed hydrogen bonds with oxygen atoms in hydroxyl group of cholesterol. This made the peptide backbone prone to be perpendicular to the hydrophobic surface formed by cholesterol molecules. The ribbon of Aβ42 peptide is displayed in orange.

In the mutant system, we analyzed the contacts between mutated Ser19 and cholesterol molecules. Again, stable contacts were recognized, but the substance of the interaction was hydrogen bond (**Figure 5**). The hydrogen atoms in amine group and alcohol group of serine formed hydrogen bonds with oxygen atoms in hydroxyl group of cholesterol. This made the peptide backbone prone to be perpendicular to the hydrophobic surface formed by cholesterol molecules.

Considering these two kinds of cholesterol-peptide interactions and different extents of β-sheet structure formation, we infer that the hydrophobic surface formed by cholesterol molecules may have served as a template and reduced the entropy of Aβ42 peptide to form β-sheet conformation.

### Sphingomyelin, DPPC and Cholesterol Affect Aβ Peptide with Different Mechanisms

**Figure 6:**
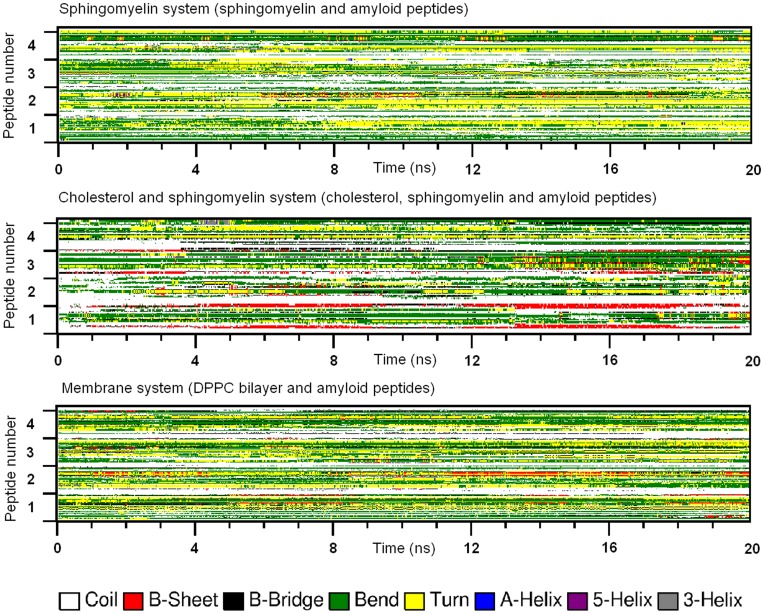
Conformational transition of Aβ42 peptides in SM system, cholesterol & SM system and membrane system. SM slightly increased the β-sheet structure, but the mixture of SM and cholesterol had a strong enhancing effect, which was similar to free cholesterol system. Meanwhile, the DPPC bilayer seemed to moderately increased β-sheet structure.

The secondary structures of Aβ42 peptides in presence of sphingomyelin (SM), SM-cholesterol mixture and DPPC bilayer are considerably different (as shown in **Figure 6** **)**. SM slightly increased the β-sheet structure, but the mixture of SM and cholesterol had a strong enhancing effect, which was similar to free cholesterol system. Meanwhile, the DPPC bilayer seemed to moderately increased β-sheet structure.

**Figure 7:**
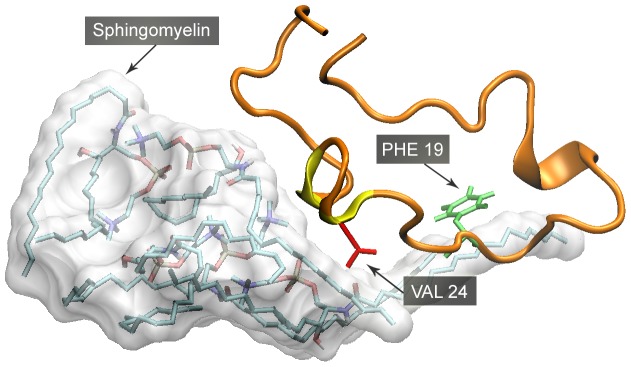
Interaction between sphingomyelin (SM) and Aβ42. It can be seen that some acyl chains of SM are exposed to water, binding with neutral residues of Aβ peptides. Phe19 (in green) is not found to stably bind to SM. But Val 24 (in red) and other neutral residues are in frequent contact with the acyl chain of SM.

The interactions of these lipids with Aβ peptides were also different. In the simulation of Sphingomyelin (SM) system (4 Aβ peptide with 12 SM molecules), separated SM molecules quickly assembled into a micelle. As some acyl chains of SM were still exposed to water, they prone to bind with natural residues of Aβ peptides. However, no stable contact between Phenylalanine and acyl chain was recognized. Instead, the residues with alkyl group tend to bind with acyl chains of SM (**Figure 7**).

**Figure 8:**
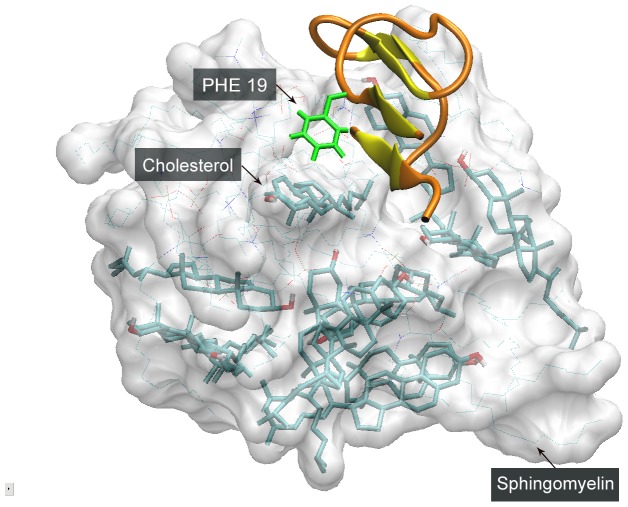
Interaction between Aβ peptide and cholesterol-SM mixed micelle. On the surface of cholesterol-SM micelle, phenylalanine (in green) bind with cholesterol, and β-sheet structure is formed adjacently.

When cholesterol and SM were simulated together, they formed a mixed micelle, with Aβ peptide stick on the surface (**Figure 8**). To clarify the binding specificity of Aβ peptide with these two types of lipids, we calculated the distances of Phe19 with cholesterol and SM. Interestingly, the mean distance of Phe19 and cholesterol (0.54 nm) was considerably shorter than that with SM (0.87 nm), suggesting a higher binding specificity Phe19 with cholesterol. This is in agreement with experimental results [14]. As cholesterol and SM are both hydrophobic lipids, our data points to the structural specificity of steroid group, rather than general hydrophobic chains, to be responsible for β-sheet induction effect.

**Figure 9:**
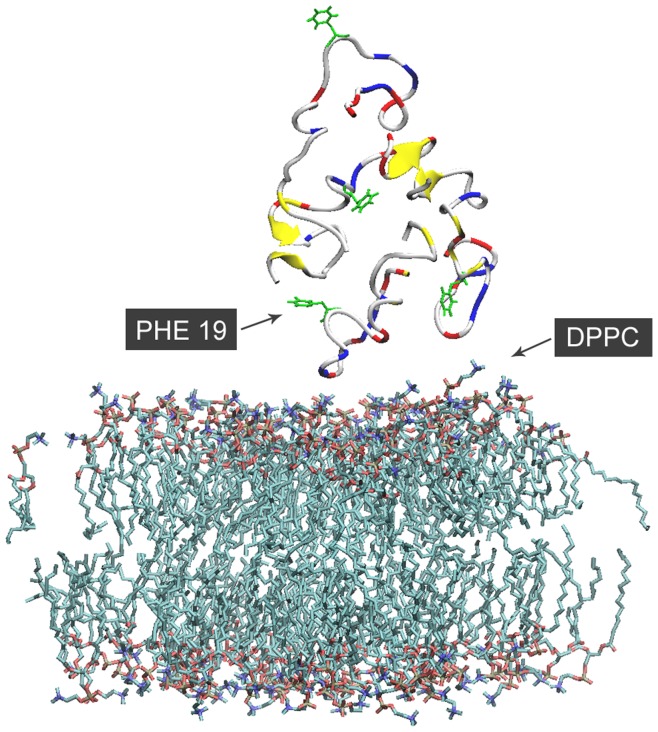
Interaction between and DPPC bilayer. Some polar residues (in red or blue) are attracted to the head groups of DPPC, and a slight deformation of the membrane was observed. On the contrary, Phe19 residues (in green) don’t contact the surface of the lipid.

In the membrane system (4 Aβ peptide with 128 DPPC), some polar residues were attracted to the head groups of DPPC, and a slight deformation of the membrane was observed (**Figure 9**). On the contrary, Phe19 residues didn’t contact the surface of the lipid. These all suggest the different substances of membrane-induced and cholesterol-induced β-sheet formation.

## Discussion

Using MD simulation method, we have studied the conformational transition of Aβ peptides in presence of cholesterol, SM and DPPC bilayer. The aromatic residue Phe19 was also mutated to examine the specificity of cholesterol interaction. Through these data, we are able to probe the mechanism of cholesterol-induced β-sheet formation.

Firstly, the interaction between Benzyl group of phenylalanine and steroid group of cholesterol plays an important role in cholesterol-induced β-sheet formation. Our simulation revealed a regular 1 ns time interval between the establishment of cholesterol-phenylalanine contact and consequent β-sheet formation. The simulated distance between Benzyl group and steroid group (0.3 nm) was also in agreement with experimental measurement [40], [41], [42]. The interaction resembles a typical mode of π-π stacking, wherein aromatic rings attract each other to form stable noncovalent binding. When cholesterol was substituted with SM, much less β-sheet structure was formed. Also, a higher affinity between Phe19 and cholesterol was found on the surface of cholesterol-SM micelle. When we made a Phe19→Ser mutation, the β-sheet formation was dramatically reduced. Based on all these results, we can conclude that the cholesterol-phenylalanine interaction is an important event in cholesterol-induced β-sheet formation.

Secondly, free cholesterol molecules form micelles that has larger flat surface with steroid group exposed. This structure may have served as a guide template for Aβ peptide to form β-sheet structure. As the carboxyl-terminal 28–40 residues make up a richly hydrophobic domain, multiple peptides tend to form amorphous aggregation. There are energy and entropy barriers for the randomly associated peptides to form β-sheet structure. However, when we simulated Aβ peptides and free cholesterol together, the peptides bound to the surface of cholesterol micelle. Comparing to the other regions of the Aβ peptide, the carboxyl terminus had a higher tendency to interact with cholesterol and to form β-sheet conformation. In fact, previous experimental study has suggested that the C-terminus of Aβ peptide forms β-sheet conformation in aqueous environment and has a stronger ability to induce aggregation than the N-terminus [43]. In the MD simulation, the flat surface exposing steroid group seemed to attract the aromatic side chains of Aβ peptide, which may be beneficial for overcoming the energy and entropy barriers to form β-sheet structure.

Furthermore, the simulations of Aβ peptides with DPPC bilayer seem to reflect another mechanism, which is based on the polar side chains of peptides and head groups of lipids. Although this mechanism is still unclear, several factors are under consideration, e.g., increase of the local concentration of the peptide upon its membrane binding, and aggregation-favoring orientation of the bound peptide [44].

We used atomic detail models to simulate Aβ peptide in presence of different lipids, and found that cholesterol induces higher β-sheet content in the Aβ peptide oligomers, which may lead to faster fibril formation. The data suggest that cholesterol may provide a flat hydrophobic surface that attracts Aβ peptide and induce β-sheet arrangement. Since cholesterol has been found enriched in senile plaques (the exact site where Aβ amyloid is deposited), the model suggested by our study is highly relevant to the pathogenesis process of Alzheimer’s disease. The increased concentration of cholesterol in the brain (both inside and outside the neurons) provides considerable chance for the interaction between cholesterol and Aβ peptides. The MD simulation may thus reflect an early phase of cholesterol-Aβ peptide interaction, which is marked by the increase of β-sheet conformation of Aβ. Due to the long time needed for amyloidal fibrils formation in vivo, we plan to scale up the simulation time in future studies. It would also be meaningful to simulate the surface of cholesterol in crystalline state and investigate its effect on Aβ peptide.

In addition to the model that our study has suggested, other mechanisms for the effect of cholesterol-Aβ interaction should also be considered, for example cholesterol may act as a seeding agent to induce Aβ oligomer growth. To address this question, MD simulations of multiple Aβ peptides and different sizes of cholesterol micelles should be performed. These efforts will help to understand the relationship between cholesterol metabolic disorder and Alzheimer’s disease.
